# Sex-dependent changes in the louse abundance of red-footed falcons (*Falco vespertinus*)

**DOI:** 10.1007/s00436-020-06634-2

**Published:** 2020-03-16

**Authors:** Imre Sándor Piross, Szablocs Solt, Éva Horváth, László Kotymán, Péter Palatitz, Péter Bertók, Krisztián Szabó, Nóra Vili, Zoltán Vas, Lajos Rózsa, Andrea Harnos, Péter Fehérvári

**Affiliations:** 1 Department of Biomathematics and Informatics, University of Veterinary Medicine, Budapest, Hungary; 2 Balaton Limnological Institute, MTA Centre for Ecological Research, Tihany, Hungary; 3 MME BirdLife Hungary, Red-footed Falcon Workgroup, Budapest, Hungary; 4 Hódmezővásárhely, Hungary; 5 Budapest, Hungary; 6 Conservation Genetics Research Group, Department of Ecology, Institute for Biology, University of Veterinary Medicine, Budapest, Hungary; 7 Hungarian Natural History Museum, Budapest, Hungary; 8 GINOP Evolutionary Systems Research Group, Institute of Evolution, MTA Centre for Ecological Research, Tihany, Hungary

**Keywords:** Phthiraptera, Amblycera, Ischnocera, Ectoparasite, Falconidae, Vertical transmission, Sex-biased infestation, Ecology

## Abstract

Permanent ectoparasites live in stable environments; thus, their population dynamics are mostly adapted to changes in the host life cycle. We aimed to investigate how static and dynamic traits of red-footed falcons interplay with the dynamics of their louse subpopulations during breeding and how they affect the colonisation of new hosts by lice. We sampled red-footed falcon (*Falco vespertinus*) nestlings (two breeding seasons) and adults (one breeding season) in southern Hungary. The mean abundance of *Colpocephalum subzerafae* and *Degeeriella rufa* lice on the nestlings was modelled with generalized linear mixed models using clutch size and host sex in interaction with wing length. For adults, we used wing length and the number of days after laying the first egg, both in interaction with sex. *D. rufa* abundances increased with the nestlings’ wing length. In one year, this trend was steeper on females. In adult birds, both louse species exhibited higher abundances on females at the beginning, but it decreased subsequently through the breeding season. Contrarily, abundances were constantly low on adult males. Apparently, *D. rufa* postpones transmission until nestlings develop juvenile plumage and choose the more feathered individual among siblings. The sexual difference in the observed abundance could either be caused by the different plumage, or by the females’ preference for less parasitized males. Moreover, females likely have more time to preen during the incubation period, lowering their louse burdens. Thus, sex-biased infestation levels likely arise due to parasite preferences in the nestlings and host behavioural processes in the adult falcons.

**Electronic supplementary material:**

The online version of this article (10.1007/s00436-020-06634-2) contains supplementary material, which is available to authorized users.

## Introduction

The abundance of ectoparasites can vary markedly between individuals, and a considerable part of this variation can be explained by certain individual host traits. Both static and dynamic host traits can influence infestation levels, like sex, age, body size and behaviour. Considering both trait types simultaneously in parasite ecological studies could help to understand the biology of host-ectoparasite interactions and explain their dynamics. Avian lice (Phthiraptera: Ischnocera, Amblycera) are permanent ectoparasites that are relatively easy to observe, collect and quantify, while their hosts constitute one of the most intensively studied taxa in ecology. Therefore, the relationship between birds and lice offers an ideal model system for parasite ecological studies.

Body size is one of the most relevant characteristics of host organisms; larger birds tend to harbour more lice in comparison to both within (Galloway and Lamb 2017; Chu et al. 2019; but see Darolova et al. 2001) and across species (Rózsa 1997; Harnos et al. 2017). Larger hosts probably provide more durable ‘habitat patches’, with larger surface areas, and more diverse sets of topographic refugia (Rózsa 1997; Poulin 2011) for lice. Since it is easy to measure, wing length is often used as a proxy of body size when examining nestlings under field conditions.

Sex has also frequently been associated with louse abundance with contradictory overall results. In some cases, the males (Rivera-Parra et al. 2014; Durkin et al. 2015), in others the females (Potti and Merino 1995; Brooke 2010), were more heavily infested, while other studies found no evidence for bias (Touleshkov 1965; Kettle 1983).

It has been recognised that louse abundance can change dynamically throughout the year but based on the existing studies, it is difficult to find clear associations with the life cycle stages of the host. Foster (1969) found that the amblyceran lice of the Orange-crowned Warbler (*Leiothlypis celata* (Say, 1822)) timed their breeding to match the host’s breeding period. Lamb and Galloway (2016) revealed that woodpecker lice breed throughout the year, but their prevalence and intensity were the lowest at the end of the breeding period. On the other hand, both Galloway and Lamb (2015) and Kettle (1983) showed that different species of lice on the same host can exhibit distinct seasonal patterns, and none of these match the reproductive cycle of the hosts. Moulting was also hypothesised to affect louse abundances (Foster 1969; Kettle 1983); however, recent field studies (Galloway and Lamb 2015) and experiments (Moyer et al. 2002) failed to find evidence supporting this notion. Although the relationship of louse population dynamics and the breeding period of the birds remained unclear, this life cycle stage has a profound impact on the birds’ physiology and behaviour which could change their ability to combat lice.

Avian lice heavily rely on vertical transmission routes from parents to offspring (Clayton and Tompkins 1995) and, therefore, their dispersion opportunities mostly open up during the host breeding season. Lice infesting nestlings in a brood have the opportunity to prefer particular siblings against others, due to the direct bodily contacts among siblings. This period offers opportunities even for lice with poor transmission capabilities to choose the most appropriate host, taking multiple host characters like body size, plumage development and sex into account. The louse load of a particular nestling, therefore, depends substantially on its individual characters as compared to its nestmates’ number and their characters. Richner and Heeb (1995) introduced the so-called dilution hypothesis, claiming that ectoparasites like lice can disperse to more nestlings in larger broods, lowering the average abundance of individuals. This pattern was recently observed by Piross et al. (2018) in Common Kestrel (*Falco tinnunculus* Linnaeus, 1758) nestlings. This effect may be accentuated by the individual differences among parents: birds in poor condition likely produce fewer, but more infested offspring than those in good condition (Whiteman and Parker 2004).

Red-footed falcons (*Falco vespertinus* Linnaeus, 1766) are colonially breeding raptors showing marked sexual dimorphism in adult plumage (absent in juvenile plumage) and different sex roles during breeding. Their habitat use and behaviour in the breeding period has been broadly studied (Palatitz et al. 2018). These falcons typically raise 3–4 nestlings of similar age (Solt 2018a) and host relatively high lice loads (Piross et al. 2015). Siblings of the same clutch tend to be genetically similar, receive similar parental care and have an equal chance to contract lice from their parents and each other. Studying the ectoparasite-host relationship in this system, therefore, allows us to rule out known influential factors such as large-scale environmental differences, and also allows focusing on the effects of individual host traits.

The aim of this work was to investigate how certain host characteristics affect the abundance of lice on both nestling and adult red-footed falcons during the breeding period. We examined the role of body size, sex, time elapsed since the commencement of breeding (in case of adults only) and clutch size (in case of nestlings only). Since we hypothesised that the difference in louse abundance between siblings may be caused by the preference of lice for either host sex, we aimed to sample clutches where both male and female nestlings were present.

## Materials and methods

### Sampling

Red-footed falcon louse infestation samples were collected in Vásárhelyi-puszta, an area within the municipality borders of Hódmezővásárhely, Békéssámson, Székkutas, Orosháza and Kardoskút (N 46°28′25″, E 20°37′30″), belonging to the Körös-Maros National Park, Hungary. This landscape is dominated by alkaline grasslands interspersed with arable fields, temporary saline lakes or marshes, farms and dirt roads. In this area, most red-footed falcons breed in artificial nest-boxes fixed on trees, but a minority of them occupy natural nest-sites built by Corvids. They readily breed colonially, but solitary breeding is also frequent (Kotymán et al. 2015). The breeding performance of the local population is closely monitored, and the vast majority of nestlings is ringed with individual colour rings and weighed and measured according to a standard protocol (Palatitz 2018). This entails body mass and wing length measurements. The latter was used as a proxy to body size.

In 2012, two (in one case three) nestlings (*N* = 95)—a presumed male and a presumed female (Ristow 2003)—from each clutch were sampled for lice (*N* = 67). In 2014, entire clutches (87 nestlings from 32 clutches), together with adult birds (*N* = 60), were sampled. The sampled clutches and adults were selected to be independent of each other to avoid cross contamination caused by the ectoparasite collection method (see below for details). Adults were trapped in the vicinity of their nests using mist nets and were colour ringed. All focal nests were subsequently revisited and observed at least twice on separate days to observe and identify colour-ringed individuals associated with the clutch. An adult bird was considered as the social parent of the clutch if some sort of parental care (incubation, feeding, etc.) was observed on both occasions. Nest-boxes were monitored 6–12 times throughout the breeding season allowing to pin-point egg laying dates to a 24-h precision (see Kotymán et al. 2015 for further details).

Dust-ruffling (Clayton and Drown 2001) was used to remove the lice from the hosts. The plumage was treated with pyrethrin powder and the birds held over a white tray for 5 min. Lice falling off were collected into a centrifuge tube containing 70% ethanol. After 5 min, the plumage was gently ruffled to dislodge the remaining parasites. The identification of lice was based on Price et al. (2003), using a stereoscopic microscope (Zeiss Stemi DRC).

### Molecular sexing of the nestlings

The sex of the nestlings was determined by molecular methods. For this purpose, three developing feathers were plucked from the back during the ringing procedure. The samples were stored in absolute ethanol at − 20 °C until further use. The feather samples were analysed in the molecular laboratory of the Institute of Biology, University of Veterinary Medicine Budapest. Total genomic DNA was extracted from the feather shaft using NucleoSpin Tissue Kit (Macherey-Nagel). Sex was determined by amplifying the CHD1-W and CHD1-Z gene introns, using the 2550F and 2718R primer pair (Fridolfsson and Ellegren 1999). To verify the molecular sexing results, two methods were used: first, another intronic part of the CHD1 gene was parallel amplified using the primer pair (CHD1-i16F and CHD1-i16R; Suh et al. 2011) in a subset of samples (*N* = 10). Second, 18 adult birds with known sex were additionally analysed. Both primer pairs gave congruent results, and sex determined by molecular analysis agreed with adult phenotypic sex in each case. PCR reactions were performed using the conditions as described by the authors publishing the primers (Fridolfsson and Ellegren 1999; Suh et al. 2011). PCR products were evaluated by agarose gel-electrophoresis.

### Statistical methods

We used generalized mixed models with negative binomial distribution and log-link (Zuur et al. 2009) to evaluate the effect of the explanatory variables on the abundance of *Colpocephalum subzerafae* Tendeiro, 1988b and *Degeeriella rufa* (Burmeister, 1838) respectively. In the case of nestlings, we analysed the samples from 2012 and 2014 separately. This was necessary for two reasons. First, different sampling procedures were implemented in the 2 years. Second, the distribution of lice differed considerably between years (Piross et al. 2015) thus incorporating both years into a single model that fits the data was infeasible. In addition, the effect of host sex by comparing siblings was evaluated. In order to do this correctly, we only used data from nestlings where we sampled both males and females in the same clutch. We hypothesised that the difference in louse abundance between siblings of different sex was caused by the preference of lice for male versus female host, and this is possible only if both sexes are present in the clutch. In adults, observations with missing values in the explanatory variables were removed. We centred (subtracted the mean) of every continuous variable before modelling to improve model fit. We used deviance-ratio tests for model selection. We removed the explanatory variables from our initial models one-by-one, and if the two models (with and without the variable) did not differ significantly (α = 0.05), we excluded the variable from the model. For the nestlings, the fixed variables for the initial models were clutch size and sex (as categorical variables), wing length (mm) and its interaction with sex. We used the clutch ID as a random factor. This approach allows to measure within clutch effects of variables such as sex. For the adults, the fixed variables were sex, wing length (mm), their interaction, the number of days after the first egg was laid, its interaction with sex, and we used the breeding colony as a random factor.

For all analyses and figures, we used R 3.6.1 (R Core Team 2019) and the ggplot2 3.2.0 (Wickham 2016), glmmTMB 0.2.3 (Brooks et al. 2017), gridExtra 2.3 (Auguie 2017), lsmeans 2.30–0 (Lenth 2016) and the RcmdrMisc 2.5–1 (Fox 2018) packages.

## Results

Three louse species were found on the red-footed falcons. *Colpocehalum subzerafae* and *Degeeriella rufa* are associated with this host species in the latest world checklist (Price et al. 2003), and the scarce occurrence of *Laemobothrion* (*Laemobothrion*) *tinnunculi* Linnaeus, 1758 was also reported in Piross et al. (2015). *L. tinnunculi* was found only on two adult birds. We have not found any *Nosopon lucidum* (Rudow 1869a) in our samples (Price et al. 2003). The descriptive statistics (Reiczigel et al. 2019) of *C. subzerafae* and *D. rufa* infestation are presented in Table 1.

**Table 1:** Descriptive statistics of the louse infestation of the red-footed falcons (*Falco vespertinus*) by age, louse species, year and sex

Age	Louse species	Year	Sex	Infected	Hosts	Prevalence	Mean abundance	Median abundance	Mean intensity	Median intensity	Variance/mean
Nestlings	*C. subzerafae*	2012	Male	34	42	81%	5.5	3	6.7	5	8.6
			Female	41	51	80%	5.9	5	7.4	7	6.9
			All	75	95	79%	5.6	4	7.1	6	7.7
		2014	Male	14	38	37%	1.1	0	3.0	2	5.3
			Female	15	46	33%	0.9	0	2.7	2	5.1
			All	29	87	33%	1.0	0	2.9	2	5.2
	*D. rufa*	2012	Male	34	42	81%	3.2	2	4.0	2	3.5
			Female	40	51	78%	2.7	2	3.4	2	3.2
			All	74	95	78%	2.8	2	3.7	2	3.4
		2014	Male	22	38	58%	2.2	1	3.9	2	6.3
			Female	31	46	67%	1.9	2	2.9	2	2.9
			All	53	87	61%	2.0	1	3.3	2	4.6
Adults	*C. subzerafae*	2014	Male	8	33	24%	4.2	0	17.4	7	55.2
			Female	4	27	15%	0.2	0	1.5	2	1.5
			All	12	60	20%	2.4	0	12.1	4	53.9
	*D. rufa*		Male	22	33	67%	6.1	1	9.1	4	19.8
			Female	12	27	44%	1.0	0	2.2	2	1.5
			All	34	60	57%	3.8	1	6.7	2	19.1

Our models indicate that louse abundance on nestlings was affected by their sex and wing length. In 2012, a marginally significant (slightly above the prescribed level of significance, *p* = 0.0565) but relevant difference was found in the abundance of *C. subzerafae* between male and female siblings. Estimated abundance was 3.5 (95% C.I. 1.7–7.2) on females compared to 2.1 (95% C.I. 1.0–4.4) on their male siblings. In 2014, none of the investigated variables had any significant effect on *C. subzerafae* abundance (see the Supplementary Material).

**Fig. 1:**
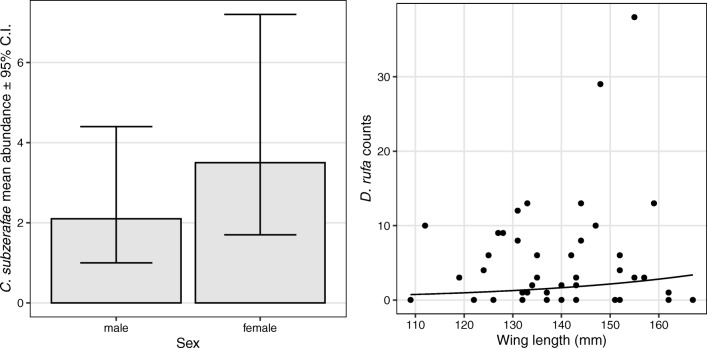
Results of the GLMMs modelling the mean abundance of the louse species on the red-footed falcon (*Falco vespertinus*) nestlings in 2012. In the case of the *Colpocephalum subzerafae*, a non-significant difference can be seen between the two sexes. In the case of *Degeeriella rufa*, the mean abundance increases with the wing length of the nestlings

**Fig. 2:**
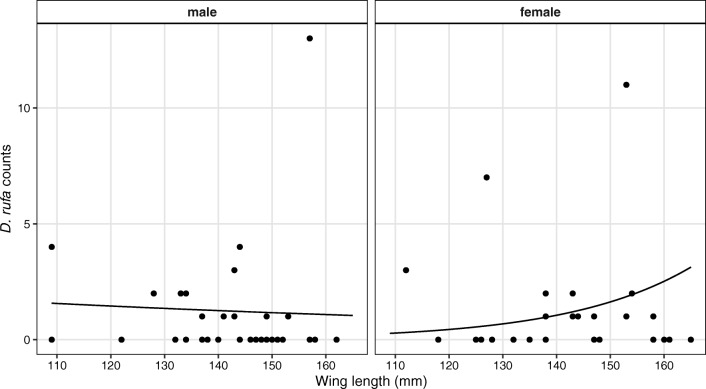
Results of the GLMM modelling the mean abundance of *Degeeriella rufa* on the red-footed falcon (*Falco vespertinus*) nestlings in 2014. There is an interaction between sex and wing length. The mean abundance increases with wing length in the case of the females

In 2012, only wing length (*p* = 0.0482), and in 2014, sex, wing length and their interaction had a significant effect (*p* = 0.0387) on the nestlings’ *D. rufa* abundance. The predicted *D. rufa* abundance was 0.7 (95% C.I. 0.2–2.1) on the smallest nestlings (wing length = 109 mm) and 3.4 (95% C.I. 1.6–7.2) on the largest ones (wing length = 167 mm). In 2014, the predicted abundance for the smallest male nestlings (wing length = 109 mm) was 1.6 (95% C.I. 0.4–5.9) and 0.3 on females (95% C.I. 0.1–1.4). On the largest male nestlings (wing length = 167 mm), it was 1.0 (95% C.I. 0.3–3.3) and 3.4 (95% C.I. 1.2–9.6) on the females. This shows that wing length has a different effect on the louse abundance depending on sex. See Table 2, Figs. 1 and 2 and the Supplementary Material for further details.

**Table 2:** Abundances (and their 95% C.I.) of the different louse species on the red-footed falcon (*Falco vespertinus*) nestlings predicted by the GLMMs

Data	Wing length (mm)		Sex	Abundance estimate	95% C.I.	
*C. subzerafae*, nestlings 2012			Female	3.5	1.7	7.2
			Male	2.1	1.0	4.4
*D. rufa*, nestlings 2012	Minimum	109		0.7	0.2	2.1
	1st quartile	133		1.4	0.8	2.4
	Median	143		1.8	1.1	2.8
	3rd quartile	152		2.3	1.4	3.7
	Maximum	167		3.4	1.6	7.2
*D. rufa*, nestlings 2014	Minimum	109	Female	0.3	0.1	1.4
	1st quartile	133		0.8	0.4	1.7
	Median	143		1.2	0.7	2.1
	3rd quartile	152		1.8	1.0	3.3
	Maximum	167		3.4	1.2	9.6
	Minimum	109	Male	1.6	0.4	5.9
	1st quartile	133		1.3	0.7	2.4
	Median	143		1.2	0.7	2.1
	3rd quartile	152		1.2	0.6	2.3
	Maximum	167		1.0	0.3	3.3

Both louse species showed a similar abundance pattern on adult falcons. Sex, the number of days elapsed since laying the first egg and the interaction of these two variables all had a significant effect on the abundance of both *C. subzerafae* (*p* = 0.0361) and *D. rufa* (*p* < 0.0001). In general, males showed low and nearly constant level of abundance as compared to females in both species. In the earlier days of breeding (day 11), predicted *C. subzerafae* abundance was 0.4 (95% C.I. 0.1–3.6) and *D. rufa* abundance was 2.0 (95% C.I. 0.8–5.0). In later days (day 54), it was 0.1 (95% C.I. 0.0–1.4) for *C. subzerafae* and 0.6 (95% C.I. 0.2–1.7) for *D. rufa*.

Females showed higher abundances in the early days that rapidly declined during the breeding period. At day 11, the predicted abundance for *C. subzerafae* was 8.5 (95% C.I. 0.8–84.8), and for *D. rufa*, it was 25.0 (95% C.I. 11.7–53.6), while at day 54, it was 0 (95% C.I. 0.0–0.5) for *C. subzerafae* and 0.2 (95% C.I. 0.1–0.6) for *D. rufa*. See Table 3, Fig. 2 and the Supplementary Material for further details. The results of the deviance tests and the AIC and BIC values of the models are available in the Supplementary Material.

**Table 3:** Abundances (and their 95% C.I.) of the different louse species on the adult red-footed falcons (*Falco vespertinus*) predicted by the GLMMs

Data	Days after first egg laid		Sex	Abundance estimate	95% C.I.	
*C. subzerafae*, adults 2014	Minimum	11	Male	0.4	0.1	3.6
	1st quartile	19		0.3	0.1	1.8
	Median	30		0.2	0.1	0.9
	3rd quartile	48		0.1	0.0	1.1
	Maximum	54		0.1	0.0	1.4
	Minimum	11	Female	8.5	0.8	84.8
	1st quartile	19		2.5	0.6	11.4
	Median	30		0.5	0.1	1.7
	3rd quartile	48		0.0	0.0	0.6
	Maximum	54		0.0	0.0	0.5
*D. rufa*, adults 2014	Minimum	11	Male	2.0	0.8	5.0
	1st quartile	19		1.6	0.8	3.2
	Median	30		1.1	0.7	2.0
	3rd quartile	48		0.7	0.3	1.6
	Maximum	54		0.6	0.2	1.7
	Minimum	11	Female	25.0	11.7	53.6
	1st quartile	19		10.1	6.0	16.9
	Median	30		2.9	1.8	4.5
	3rd quartile	48		0.4	0.1	1.0
	Maximum	54		0.2	0.1	0.6

## Discussion

In the present work, we showed that the two examined louse species can exhibit sex-biased infestation patterns that are interconnected with different host traits across different life-history stages. Our generalized mixed-effect modelling approach enabled us to take several factors and their interactions into account, revealing the invading parasites’ preference for female nestlings and possibly show examples of both parasite- and host-mediated sex-biased infestation.

Wing length (a proxy of body size) exhibited noticeable effects on the abundance of lice infesting nestlings in case of *D. rufa*, while *C. subzerafae* was seemingly unaffected by the size of the nestlings (see Table 2; Figs. 1 and 2). These two lice belong to different suborders that exhibit different evasion mechanisms to reduce mortality due to host defences. Amblycerans, like *C. subzerafae*, often avoid preening by running swiftly in the plumage, and also on the skin surface. Contrarily, ischnocerans—like *D. rufa*—can hide and attach themselves to particular topographic refugia in the host plumage (Johnson and Clayton 2003). The two suborders tend to rely on different diets as well. Ischnocerans mostly graze feather barbules, while amblycerans also chew skin fragments and consume blood. Consequently, ischnocerans probably rely more heavily on host plumage than amblycerans (Johnson and Clayton 2003). Our findings corroborate this hypothesis as *D. rufa* possibly postpones infestation of nestlings until the juvenile plumage is well developed.

Nestlings’ sex also affects louse abundances to some extent. In one of the two study years, we found weak evidence that female nestlings harbour ~ 60% more on average of *C. subzerafae* than their male siblings (see Table 2 and Fig. 1). In 2014—but not in 2012—an increasing trend of *D. rufa* abundance was also detected on female nestlings, but not on males (see Table 1 and Fig. 2). Since the nestling period is not much longer than the typical generation time of avian lice (Johnson and Clayton 2003), we assume that this increase was not caused by the multiplication of lice on falcon nestlings; rather, they represented the influx of lice transmitted from the parents. This implies that the sexual differences in nestling infestation levels are probably caused by parasite preferences for female hosts. As far as the nestlings stay in the nest, their bodily contacts enable lice to move freely across and probe all members of the clutch providing ample time and opportunity for individual lice to decide on their final host.

An alternative to this hypothesis would be to presume that male and female nestlings are equally infested, but their antiparasitic defences are different. The most important avian defences against lice are preening and grooming (Clayton et al. 2010), with the immune response also involved in the case of amblyceran lice (Møller and Rózsa 2005). In this case, however, we would expect the female sex to exhibit more effective defences and lower infestation rates (Zuk and McKean 1996; Poulin 1996), opposite to the phenomenon documented above.

Further, red-footed falcons at this age hardly show any morphological dimorphism aside from that females tend to be slightly larger, but body size (measured as wing length) was taken into account in our statistical procedure. Therefore, there is no apparent explanation of why lice should prefer female nestlings over their male siblings in the short term. In the long term, arguably, lice on female red-footed falcons may achieve higher fitness due to their ability to establish larger subpopulations on adult females. Similar results were found on the closely related (Fuchs et al. 2015) Amur falcons *Falco amurensis* Radde, 1863, where *D. rufa* abundances were higher on adult females than adult males (Piross et al. 2019). This assumes a behavioural adaptation of lice: they need to make adaptive decisions on which host individual to choose in the nest.

In 2014, the nestlings were less infested (see Table 1; Piross et al. 2015) with both louse species, which may explain why we found different patterns in the 2 years. Considering *C. subzerafae*, the evidence for sex-biased infestation was weak in 2012. Since the lice were scarcer in 2014, a similarly subtle slight difference—if present—was impossible to detect.

**Fig. 3:**
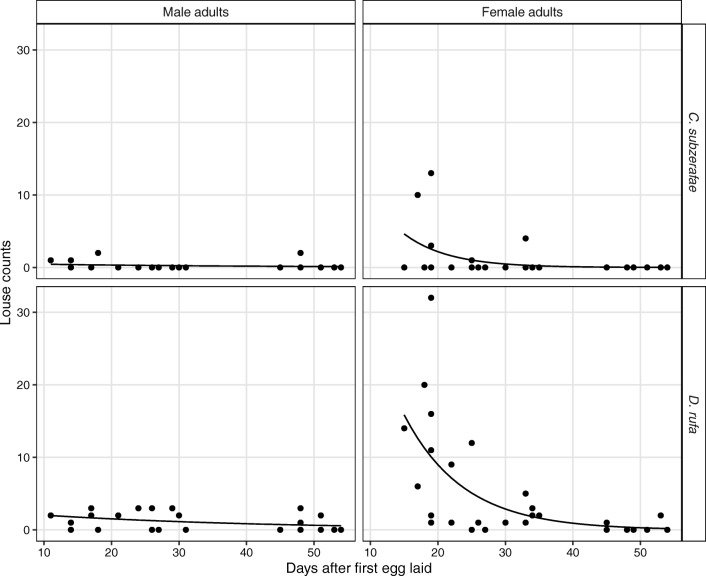
Results of the GLMMs modelling the mean abundance of the louse species on the adult red-footed falcons (*Falco vespertinus*) in 2014. There is an interaction between sex and the number of days after the first egg was laid in both louse species. The mean abundance of lice decreases with the number of days passed on female birds, while males maintain a low abundance level

On the adult birds, louse abundances show complex temporal dynamics in interaction with sex. While male birds tend to maintain a nearly constant, low level of abundance for both investigated species, females’ initially higher louse abundances decrease over the breeding period (see Table 3 and Fig. 3). Although it would be tempting to assume that the lice transferring from mothers to the offspring are causing this phenomenon, there is a temporal mismatch between the increase in *D. rufa* abundance on the nestlings and the decrease in louse abundance in general on the females. Red-footed falcon nestlings hatch 28 days after the first egg was laid (Solt 2018a). By this time, the abundance of both louse species has been already dropped (Fig. 3). It seems likely that host behavioural changes are behind this decrease. Although both sexes incubate the eggs, their behaviour differs while not incubating. Males hunt both for themselves and their mates, whereas females are more inactive and tend to rest in the vicinity of the nest (Solt 2018b). Therefore, females may allocate more time to body maintenance behaviours like preening and grooming, since they are mostly relieved from foraging during the incubation period. Possibly, this sex role differentiation in the hosts allows females to decrease their own ectoparasite load and consequently lower the future parasite load of their offspring.

It is less clear what causes the initial difference in louse abundance on adult males and females. It has long been hypothesised that parasites negatively affect the chances of males to find mates (Hamilton and Zuk 1982; and see experiment in Clayton 1990). Males may, therefore, invest more into antiparasitic behaviour prior to the commencement of breeding to increase their chances during mate choice. Furthermore, males have in general a darker, melanised plumage compared to females. Melanin was hypothesized to be less digestible for lice (Bonser 1995); thus, males may comprise less favourable habitats for lice, although this idea could not be verified in case of Rock Doves (Bush et al. 2006).

Our study has shown sexual biases in the louse infestations of red-footed falcon nestlings that show intricate patterns in interaction with other host traits. Such biases can either arise due to adaptive host-preference decisions by the parasites (i.e. parasite-mediated), or different time allocation to anti-parasite defences (i.e. host-mediated), depending on the life stage of the birds. Learning from the nestlings’ example, it is also worth exploring further whether sexually monomorphic species or life stages could exhibit sex-biased louse infestation.

## Electronic supplementary material

ESM 1 (DOCX 18 kb)
