# Supplementary material for: Sex-dependent changes in the louse abundance of red-footed falcons (Falco vespertinus)
Source: Parasitol Res. 2020 Mar 16;119(4):1327–35. doi: 10.1007/s00436-020-06634-2 (PMC7176593; doi:10.1007/s00436-020-06634-2)
Supplement: Supplementary file 1 — (DOCX 18 kb) [file 436_2020_6634_MOESM1_ESM.docx]

**Supplementary Material**

The following tables contain information on each generalized linear model, for each dataset used. The fix effects can be separated by the following operators: “+”: no interaction between the variables, “:”: the interaction variable of the two variables, “*”: the variables and their interaction variable.

Abbreviations:

d.f: The model’s degree of freedom

AIC: Akaike’s Information Criterion

BIC: Bayesian Information Criterion

χ^2^: Chi-squared value

χ^2^ d.f.: Chi-squared test’s degree of freedom

*C. subzerafae*: *Colpocephalum subzerafae* Tendeiro, 1988b

*D. rufa*: *Degeeriella rufa* Burmeister, 1838

| Data | Fix effects | d.f. | AIC | BIC | Log-L. | Deviance | χ^2^ | χ^2^ d.f. | p-value |
| --- | --- | --- | --- | --- | --- | --- | --- | --- | --- |
| C. subzerafae, nestlings 2012 | Without fix effects | 3 | 232.990 | 238.280 | -113.500 | 226.990 |  |  |  |
|  | Sex | 4 | 231.360 | 238.400 | -111.680 | 223.360 | 3.640 | 1 | 0.0565 |
|  | Sex + Wing length | 5 | 233.030 | 241.840 | -111.520 | 223.030 | 0.330 | 1 | 0.5684 |
|  | Sex * Wing length | 6 | 234.960 | 245.520 | -111.480 | 222.960 | 0.080 | 1 | 0.7811 |
|  | Sex * Wing length + Number of siblings (categorical) | 8 | 236.890 | 250.980 | -110.450 | 220.890 | 2.070 | 2 | 0.3561 |
| D. rufa, nestlings 2012 | Without fix effects | 3 | 167.310 | 172.600 | -80.660 | 161.310 |  |  |  |
|  | Wing length | 4 | 165.410 | 172.450 | -78.700 | 157.410 | 3.900 | 1 | 0.0482 |
|  | Sex + Wing length | 5 | 167.290 | 176.100 | -78.650 | 157.290 | 0.120 | 1 | 0.7297 |
|  | Sex * Wing length | 6 | 169.150 | 179.720 | -78.580 | 157.150 | 0.140 | 1 | 0.7094 |
|  | Sex * Wing length + Number of siblings (categorical) | 8 | 172.670 | 186.760 | -78.340 | 156.670 | 0.480 | 2 | 0.7869 |
| C. subzerafae, nestlings 2014 | Without fix effects | 3 | 167.390 | 174.130 | -80.690 | 161.390 |  |  |  |
|  | Wing length | 4 | 169.030 | 178.030 | -80.520 | 161.030 | 0.360 | 1 | 0.551 |
|  | Sex + Wing length | 5 | 171.000 | 182.250 | -80.500 | 161.000 | 0.030 | 1 | 0.8641 |
|  | Sex * Wing length | 6 | 172.910 | 186.410 | -80.460 | 160.910 | 0.090 | 1 | 0.7634 |
|  | Sex * Wing length + Number of siblings (categorical) | 8 | 175.660 | 193.650 | -79.830 | 159.660 | 1.250 | 2 | 0.5348 |
| D. rufa, nestlings 2014 | Without fix effects | 3 | 251.330 | 258.080 | -122.670 | 245.330 |  |  |  |
|  | Wing length | 4 | 252.340 | 261.330 | -122.170 | 244.340 | 0.990 | 1 | 0.3186 |
|  | Sex + Wing length | 5 | 254.290 | 265.540 | -122.150 | 244.290 | 0.040 | 1 | 0.8397 |
|  | Sex * Wing length | 6 | 252.020 | 265.510 | -120.010 | 240.020 | 4.270 | 1 | 0.0387 |
|  | Sex * Wing length + Number of siblings (categorical) | 8 | 255.350 | 273.340 | -119.680 | 239.350 | 0.670 | 2 | 0.7157 |
| C. subzerafae, adults 2014 | Without fix effects | 3 | 94.860 | 100.830 | -44.430 | 88.860 |  |  |  |
|  | Sex * days after first egg laid | 6 | 92.320 | 104.260 | -40.160 | 80.320 | 8.540 | 3 | 0.0361 |
|  | Sex * days after first egg laid + Wing length | 7 | 92.730 | 106.650 | -39.370 | 78.730 | 1.590 | 1 | 0.2068 |
|  | Sex * days after first egg laid + Sex : Wing length | 8 | 93.340 | 109.250 | -38.670 | 77.340 | 1.390 | 1 | 0.2385 |
| D. rufa, adults 2014 | Without fix effects | 3 | 234.860 | 240.820 | -114.430 | 228.860 |  |  |  |
|  | Sex * days after first egg laid | 6 | 207.200 | 219.130 | -97.600 | 195.200 | 33.660 | 3 | <0.0001 |
|  | Sex * days after first egg laid + Wing length | 7 | 206.560 | 220.480 | -96.280 | 192.560 | 2.640 | 1 | 0.1042 |
|  | Sex * days after first egg laid + Sex : Wing length | 8 | 207.830 | 223.740 | -95.920 | 191.830 | 0.720 | 1 | 0.3951 |
